# MbT-Tool: An open-access tool based on Thermodynamic Electron Equivalents Model to obtain microbial-metabolic reactions to be used in biotechnological process

**DOI:** 10.1016/j.csbj.2016.08.001

**Published:** 2016-08-26

**Authors:** Pablo Araujo Granda, Anna Gras, Marta Ginovart

**Affiliations:** a Chemical Engineering Faculty, Central University of Ecuador, Ciudad Universitaria – Ritter s/n y Bolivia, P.O. Box. 17-01-3972, Quito, Ecuador; b Department of Agri-Food Engineering and Biotechnology, Universitat Politècnica de Catalunya, Edifici D4, Esteve Terradas 8, 08860 Castelldefels, Barcelona, Spain; c Department of Mathematics, Universitat Politència de Catalunya, Edifici D4, Esteve Terradas 8, 08860 Castelldefels, Barcelona, Spain

**Keywords:** MbT-Tool, Microbial yield prediction, Thermodynamics, Microbial metabolism, Microbial metabolic reaction, Energy-transfer-efficiency

## Abstract

Modelling cellular metabolism is a strategic factor in investigating microbial behaviour and interactions, especially for bio-technological processes. A key factor for modelling microbial activity is the calculation of nutrient amounts and products generated as a result of the microbial metabolism. Representing metabolic pathways through balanced reactions is a complex and time-consuming task for biologists, ecologists, modellers and engineers. A new computational tool to represent microbial pathways through microbial metabolic reactions (MMRs) using the approach of the Thermodynamic Electron Equivalents Model has been designed and implemented in the open-access framework NetLogo. This computational tool, called MbT-Tool (Metabolism based on Thermodynamics) can write MMRs for different microbial functional groups, such as aerobic heterotrophs, nitrifiers, denitrifiers, methanogens, sulphate reducers, sulphide oxidizers and fermenters. The MbT-Tool's code contains eighteen organic and twenty inorganic reduction-half-reactions, four N-sources (NH_4_^+^, NO_3_^−^, NO_2_^−^, N_2_) to biomass synthesis and twenty-four microbial empirical formulas, one of which can be determined by the user (C_n_H_a_O_b_N_c_). MbT-Tool is an open-source program capable of writing MMRs based on thermodynamic concepts, which are applicable in a wide range of academic research interested in designing, optimizing and modelling microbial activity without any extensive chemical, microbiological and programing experience.

## Highlights

• Represent microbial metabolism based on thermodynamics.
• Write microbial metabolic reactions to represent environmental processes.
• An open-source computational tool to study microbial activity
• The basis of metabolic behaviour-rules embedded in an individual-based model.

## Graphical abstract

Image highlights: MbT-Tool is a computational tool to represent the microbial metabolism through a microbial metabolic reactions using a thermodynamic approach based on the energy-transfer-efficiency between catabolic and anabolic processes. The MbT-Tool has been implemented in the open-source platform NetLogo, which makes this tool accessible to chemists, biologists, ecologists, engineers and modellers, among others.

**Figure:**
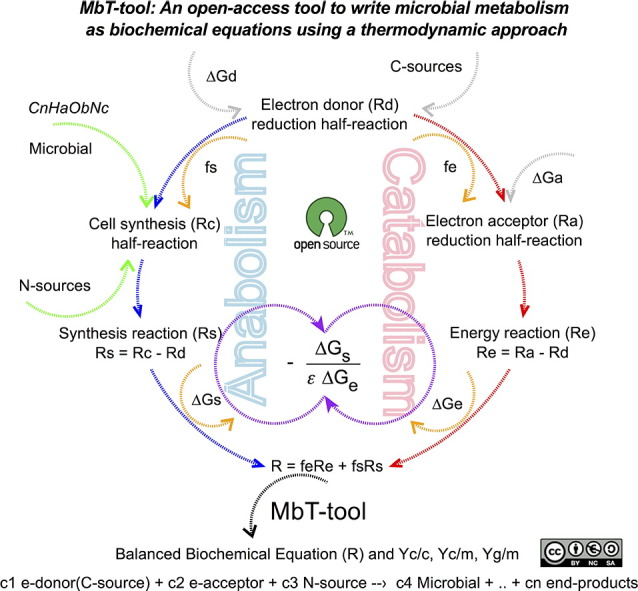

## 1 Introduction

For the construction and development of models of living organisms, it is necessary to describe their physical constants, physiological reactions, interactions and responses to the environment. A robust model of any living system must include chemicals and conservation principles, and relate stoichiometric mass balance with the laws of conservation of energy. Most biochemical processes that involve energy transduction close to the balance can be studied using a non-equilibrium thermodynamic approach. Intuitively, a complete model has a number of sub-models that take into account the compartmentalized structure of living organisms; and specifically the metabolic sub-model should include descriptions of different parts and functions of their developmental stages [1], [2], [3].

The use of microbial metabolic reactions (MMRs) that include the microbial bio-mass as a product or as a reactant is a relevant task for modelling microbial activity. An MMR is the written version of a biochemical process. The stoichiometry is a part of the chemistry that permits the writing of balanced equations because it studies the molar relation between the reactants and the products in a chemical reaction. There are several reasons to suggest that the presence of micro-organisms in a biochemical process complicates the stoichiometry. First, biochemical reactions often involve oxidation and reduction of more than one chemical species. Second, the micro-organisms have two different roles: they perform the reaction and also they are the final products of it. Third, the micro-organisms produce several chemical reactions in order to capture part of the liberated energy for cell synthesis [4], [5]. The overall procedure for representing these reactions in balanced equations is a considerable and sometimes complex task for biologists, ecologists, modellers, and engineers, among others.

The cellular metabolism has been studied, investigated and described at many levels ranging from traditional enrichment tools, molecular and genetics tools, mathematical modelling of micro-biological multispecies systems, to non-equilibrium thermodynamics [4], [6]. In the field of non-equilibrium thermodynamics, several approaches have been reported to develop a rigorous thermodynamic description for biomass yield prediction based on the energy-transfer-efficiency (*ε*) between catabolism and anabolism [4], [5], [7], [8], [9], [10], [11], [12], [13], [14], while other thermodynamic approaches correlate the biomass yield calculations with the Gibbs energy dissipations [6], [15], [16], [17], [18], [19], [20], [21]. In brief, the thermodynamic approaches explain how catabolic and anabolic reactions are coupled to predict the biomass yield in terms of standard Gibbs energy. Considering these diverse approaches in the bio-thermodynamics field, this study takes into consideration the one that is based on *ε*, because it includes: i) the standard Gibbs energy for cell synthesis from different carbon-sources and nitrogen-sources, ii) the energy available from substrate transformation, and iii) the *ε* to the overall metabolic process. In addition, choosing the thermodynamic approach allows the prediction of full stoichiometric relationships for biological reactions in the absence of experimental yield values [5], [12].

One powerful tool that is currently revolutionizing the study of microbial biotechnology is the mathematical and computing modelling that explicitly takes into account the microbial diversity and the exchanges of nutrients and materials within the micro-organism community and its surrounding environment [22], [23]. Models such as individual-based models (IBMs) [24], [25], [26], [27], metabolic energy-based modelling (EBMs) [28], reaction-centric models (RCMs) [29], and even, deterministic-population models (DPMs) [30] can obtain useful information from a MMR. The reason is that these reactions, or the stoichiometry between chemical species described in the metabolic sub-model, can help in understanding how the microbial interactions happen and why microbial systems behave as they do.

Environmental microbiologists could take advantage of the knowledge of metabolic reactions because usually the chemical resources for the micro-organisms are the pollutants that technicians must control [4]. In addition, the information provided by MMRs could be of importance in other engineering fields because they provide information about the quantities of chemicals required to satisfy microbial requirements as well as the quantities of the generated end products [5].

In this context, the principal aim of this contribution is to develop a computational tool to write MMRs for processes driven by a range of microbes based on the Thermodynamic Electron Equivalents Model (TEEM), a methodology proposed by Perry L. McCarty [4], [7], [8], [9], [11], which is the baseline of many current bio-technological studies [4], [27], [31].

The specific objectives are: (i) to implement the tool on the open-access platform NetLogo to distribute the source-code among the scientific community, (ii) to promote its use and to encourage future extensions and adaptations; and (iii) to test the computational tool by writing of some MMRs.

This manuscript presents the following structure: Section 2 describes the TEEM thermodynamic approach, the basic concepts about the empirical chemical composition used to represent the microorganisms and the open-access software used to implement the computational tool; Section 3 describes the design, performance and application of the computational tool and presents some MMRs for diverse metabolic functional groups of micro-organisms; and finally Section 4 presents some final remarks and possible future applications for this work.

## 2 Materials and Methods

### 2.1 TEEM: the thermodynamic approach

The Thermodynamic Electron Equivalents Model (TEEM) is designed to study the stoichiometry and kinetics of a wide variety of biological treatments of wastewaters. TEEM writes a stoichiometric reaction to describe the overall cellular metabolism from reduction-half-reactions for: the electron-donor (Rd), electron-acceptor (Ra) and cell synthesis (Rc). TEEM is based on terms of the standard Gibbs free energy involved in these reactions and in how the energy between catabolism and anabolism is coupled [4], [7], [8], [9], [10], [11].

For the use of TEEM, no specific and detailed knowledge of metabolism is required. First, we have to identify the electron donor(s) (eD) and the electron acceptor(s) (eA) and write reduction-half-reactions for each one of them. Second, it is necessary to establish the N-source for biomass synthesis, and third, it is essential to determine the empirical chemical formula that will represent the microbial cells.

TEEM has two versions, the first one, TEEM1 [4], considers a realistic formulation of the anabolic reaction taking into account different N-sources such as ammonium (NH_4_^+^), nitrate (NO_3_^−^), nitrite (NO_2_^−^) and di-nitrogen (N_2_), and a complete explanation of *ε* between catabolism and anabolism. The second version, TEEM2 [11], complements TEEM1 because it considers oxygenase reactions involved and the aerobic heterotrophic oxidation of C1 organic compounds.

According to TEEM, the metabolic energy is obtained from the redox reaction between an eD with an eA. The electrons are obtained from eD and transferred to intra-cellular intermediates. In TEEM1 the intermediate is the pyruvate with its half-reaction standard Gibbs free energy equal to 35.09 kJ/eeq, and in TEEM2 the intermediate is acetyl-CoA with its half-reaction standard Gibbs free energy equal to 30.9 kJ/eeq. The intermediate compounds bring the electrons towards the eA, which is being reduced causing the initial carrier regeneration [11].

TEEM calculates microbial yield (Yc/c) using a relation between the standard Gibbs free energy of the catabolic and anabolic reactions and an appropriate *ε* value. The microbial catabolism is represented by the energy reaction (Re). To write it we have to combine Rd with Ra. Once Re is known, it is necessary to represent the microbial anabolism by writing the reaction for microbial biomass synthesis (Rs), and to do this we have to combine Rd with Rc.

Rc is a hypothetical half-reaction, which considers as reactants the N-source (NH_4_^+^, NO_3_^−^, NO_2_^−^ or N_2_), carbon dioxide (CO_2_) and bicarbonate (HCO_3_^−^), and as products water and the microbial biomass represented by an empirical chemical formula of cells (C_n_H_a_O_b_N_c_). This empirical chemical formula considers the molar relationships only for four basic elements: Carbon (*n*), Hydrogen (*a*), Oxygen (*b*) and Nitrogen (*c*). To establish the adequacy of this formula, researchers compared theoretical thermodynamic calculations using the cell's empirical chemical formula with the thermodynamics of growth of the same micro-organism on several substrates using batch cultures growing in the exponential phase at μ_max_[32], [33], [34], [35]. If the formula only considers four elements, the fitness is close to 95%, but if we include two more elements, e.g. phosphorous and sulphur, the fitness increase its value to around 98–99% [36], [37].

To estimate the standard Gibbs free energy of Rc (∆G_pc_), TEEM proposes a value of 3.33 KJ per gram cells [4], [9], [11], which is related to one generic microbial cell composition C_5_H_7_O_2_N when NH_4_^+^ serves as the N-source for cell synthesis. The ∆G_pc_ value is valid in the context in which TEEM was developed. This does not make a great deal of difference in calculated microbial yields, but is felt to be an acceptable theoretical choice [11]. In other research fields, e.g. bio-geochemical processes in marine environments, and taking into account different pressure, temperature, pH and N-sources for cell synthesis the reported ∆G_pc_ value is 302 J per gram cells [3], [37], [38]. Moreover, TEEM considers in its internal structure the possibility of using four different N-sources to write Rc [4]. The theoretical explanation is that micro-organisms prefer to use ammonium as an inorganic nitrogen source for cell synthesis, because it is already in the (-III) oxidation state, the status of organic nitrogen within the cell. However, when ammonium is not available for cell synthesis, many prokaryotic cells could use oxidized forms of nitrogen as alternatives. Therefore, nitrate (NO_3_^−^), nitrite (NO_2_^−^) and di-nitrogen (N_2_) are included as nitrogen sources. When an oxidized form of nitrogen is used, micro-organisms must reduce it to the (-III) oxidation state of ammonium, a process that requires electrons and energy, thus reducing their availability for synthesis. Therefore, different N-sources will obtain different results in the microbial yield [4], [11].

To couple the energy from catabolism to anabolism, TEEM considers a relation between the electrons involved. The electrons that come from the eD will be divided into two portions. The first portion (*fe*^*o*^) is transferred to the eA to generate energy (catabolism) and the other portion of electrons (*fs*^*o*^) is transferred to the N-source for cell synthesis (anabolism). TEEM calculates the relationship between *fe*^*o*^ and *fs*^*o*^ using: (i) standard Gibbs free energy of Rd, Ra and Rc, (ii) standard Gibbs free energy of the intracellular intermediates compounds, and (iii) an appropriate *ε* value. This term is included because TEEM considers that a fraction of the thermodynamic free energy involved is lost at each energy transfer between catabolism and anabolism [13], [14].

With this information, an MMR (R = *fe*^*o*^Ra + *fs*^*o*^Rc − Rd) is written. This reaction represents the full stoichiometric relationships for a biological process without the experimental yield values commonly required to obtain it [12]. For a detailed description of this thermodynamic approach the reader can refer to McCarty (2007) and Rittmann and McCarty (2001).

### 2.2 The NetLogo platform

The computational tool is implemented in the widely used, free and open source program NetLogo, a multi-agent programmable modelling environment [39]. NetLogo is licensed under GPL (GNU General Public Licence) and it was chosen mainly for the way this platform is organized: the source-code is very well documented, open and easy to read, giving the option to share this developed tool with other researchers without difficulty. This straightforward interaction, in the near future, will facilitate the upgrading of the computational tool by the scientific community interested in writing and using metabolic reactions for microbial processes.

## 3 The developed computational tool: MbT-Tool

We have named the computational tool as MbT-Tool, standing for Metabolism based on Thermodynamics. MbT-Tool is the interactive tool developed to study and model microbial metabolism based on two versions of TEEM: TEEM1 [4] and TEEM2 [11]. With the MbT-Tool it is possible to write MMRs conducted by microbes. Moreover, using the MbT-Tool it is possible to predict the microbial yield in standard thermodynamic conditions, e.g. P = 1 bar, temperature = 25 °C and pH = 7.0.

The MbT-Tool user interface displays all variables and parameters regarding the composition of the microbial biomass and the substrates that can be selected to describe the metabolic processes, avoiding the complex and tedious calculations of the implemented thermodynamic model by the user. The composition of the microbial biomass, which is represented by C_n_H_a_O_b_N_c_, seems to be related to the substrates where the micro-organism grows and it can be slightly different depending on the C-sources and N-sources [14]. However, Battley (2013) asserts that the empirical composition of the same cells metabolizing any given substrate is expected to be the same. Therefore, the MbT-Tool considers Battley's hypothesis as valid and allows use of the same microbial biomass formula and writing MMR using different growing substrates.

**Fig. 1:**
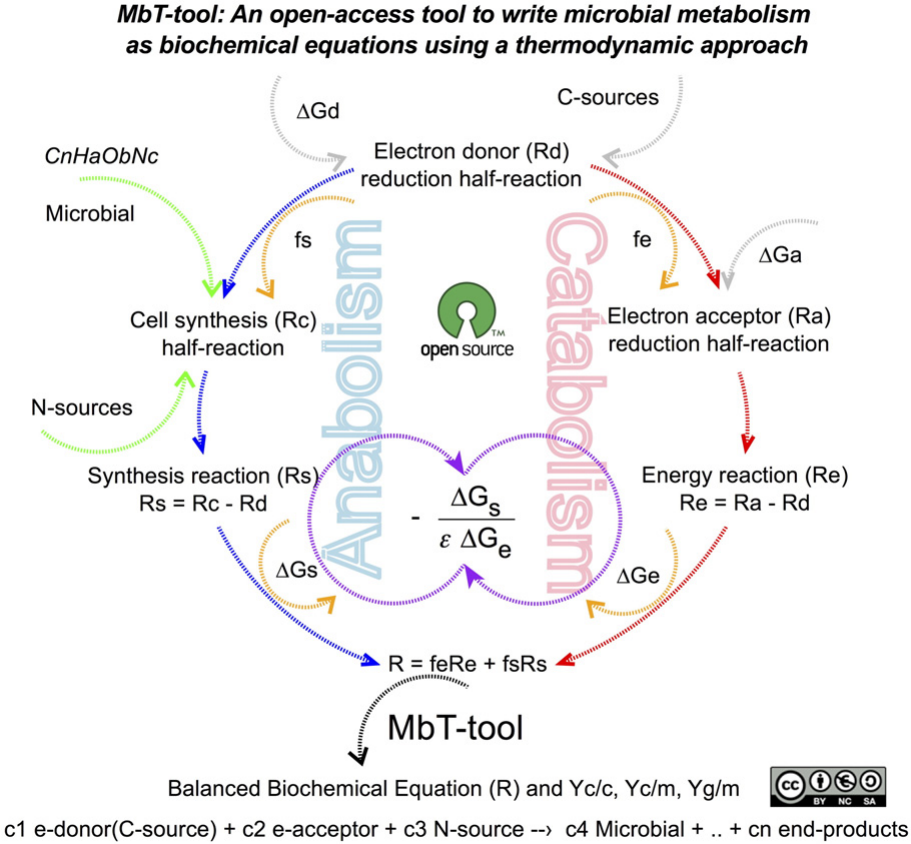
MbT-Tool is a computational tool to represent the microbial metabolism through a microbial metabolic reaction (R) using TEEM as thermodynamic approach which is based on the energy-transfer-efficiency between catabolic and anabolic processes.

**Fig. 2:**
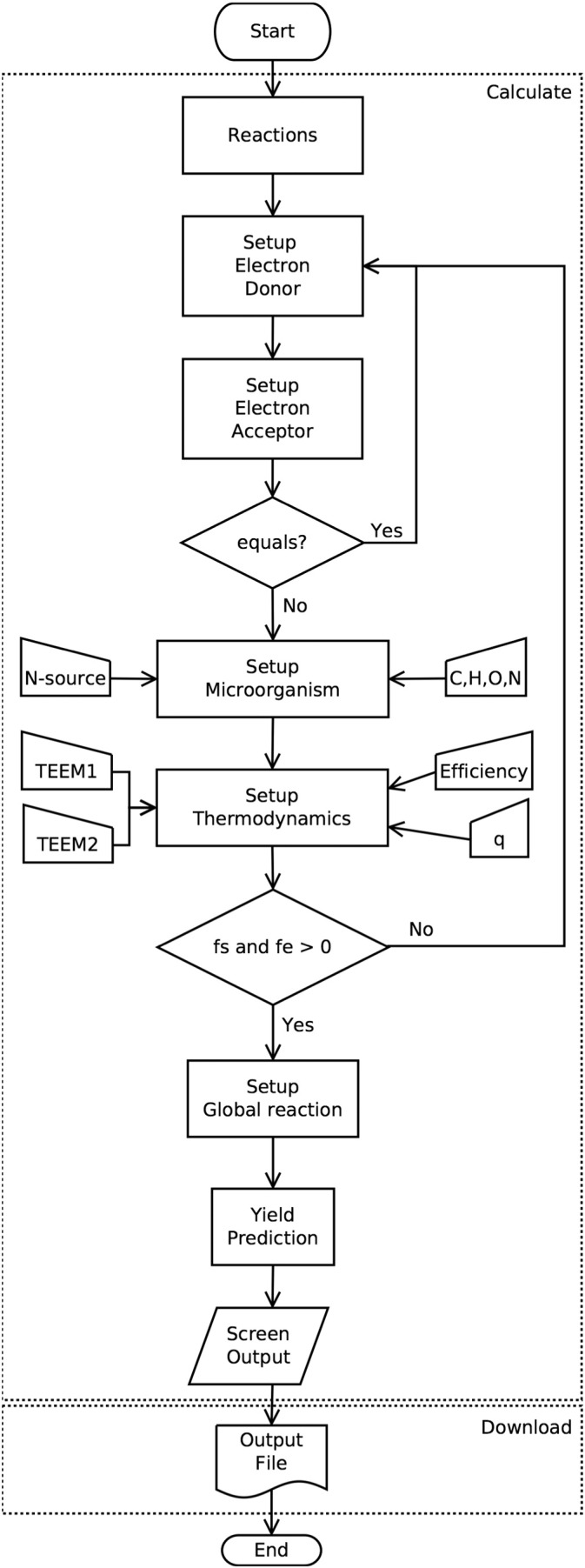
Flow chart of MbT-Tool.

**Fig. 3:**
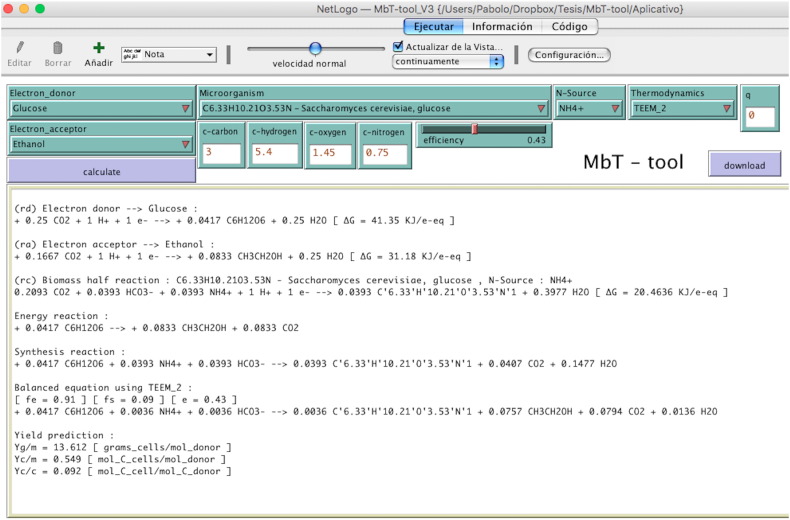
Screenshot of the MbT-Tool's user interface.

Fig. 1 shows the schematic description of how the MbT-Tool is based on TEEM with a brief explanation of TEEM concepts which are helpful in understanding the main terms involved in it, especially how energy from catabolism is coupled to anabolism and as a result of these combinations a MMR is obtained. Fig. 2 shows the schematic programming structure of MbT-Tool using a flow diagram. Fig. 3 shows a screen-print of the MbT-Tool, which includes: *interface*, *info* and *code* tabs. The *interface* tab is where the reader can obtain the outputs. It also has inputs-options that can be used to set up the MbT-Tool. The *info* tab provides an introduction to the MbT-Tool. It explains how it was created and how to use it. The *code* tab is where the code is stored. The reader can find the lists of organic and inorganic reactions, TEEM equations with theoretical half-reactions of biomass formation taking into account different N-sources, and other programmed details of the MbT-Tool in the supplementary material.

### 3.1 How to use MbT-Tool

To use the developed computational tool, it is necessary to install NetLogo (at least NetLogo 5.0) (https://ccl.northwestern.edu/netlogo/download.shtml), and download the MbT-Tool (http://mosimbio.upc.edu/en/publications/publications-by-year/years-2010-now). It runs on any computer that supports JAVA with an operating system (OS): Windows, Mac OS X and Linux [22], [39], [40].

On opening MbT-Tool, the interface window appears (Fig. 3). On the first screen the user has to select: one or two electron donor(s) and one electron acceptor from the organic (in supplementary material, Table S1) or inorganic (in supplementary material, Table S2) reduction-half-reactions, and the empirical chemical formula of the cells (in supplementary material, Table S3) or can introduce the molar relationship between the four main elements (C, H, O and N), the N-source to the cell synthesis half-reaction. It is possible to choose between four sources, NH_4_^+^, NO_3_^−^, NO_2_^−^ or N_2_ (please refer to the supplementary material, Table S4).

With this setup information, the user has to select the thermodynamic approach TEEM1 or TEEM2 (in supplementary material, Table S5) to write the MMRs. If the user chooses TEEM2 it is necessary to define the number of oxygenase reactions per mole of substrate, introducing an integer number in the “q” parameter. Finally, the user has to fix the *ε* value for the process. With all this data, the MbT-Tool displays the following outputs: the Rd, Ra and Rc half-reactions, the energy reaction (Re), the synthesis reaction (Rs), *fe*^*o*^, *fs*^*o*^, *ε*, the MMR (R) and the calculated microbial yield (Fig. 3). This MMR is the reaction that McCarty defines as global reaction, which arises from the combination between: the catabolic reaction with the anabolic reaction and the value of *ε*[11]. The user could also download these outputs in an archive with a “.txt” extension. The file name is written with the information of the Rd, Ra, Rc, N-source and the thermodynamic approach used (TEEM1 or TEEM2).

To avoid potential errors when using the MbT-Tool, we recommend not selecting the same chemical species to the eD and to the eA; they must be different. If eD and eA are the same, the result of the calculations using their standard Gibbs free energy could provoke an inconsistent value on the fraction of electrons destined for cellular synthesis or electrons destined for energy. An inconsistent value will be obtained when these fractions of electrons are greater than one or below zero. If this numerical inconsistency occurs, the MbT-Tool stops and shows an alert message to the user.

Regarding the decision of using TEEM1 or TEEM2, it all depends on: (i) the growth substrate, (ii) the microorganism(s) involved, and (iii) the metabolic pathway that will be represented using the MbT-Tool. For instance, if the growth substrate is a C1 compound, it is better to use TEEM2 over TEEM1. If one of the intermediates metabolic compounds in the pathway is pyruvate, it is better to use TEEM1 over TEEM2, If Acetyl-CoA is an intermediate compound in the pathway, it is better to use TEEM2 over TEEM1. Finally, if oxygenase reactions are involved, it is better to use TEEM2 over TEEM1. Basically, micro-organisms utilize the oxygenase reactions to create more biodegradable forms of substrates. Some examples are: when alkanes are hydroxylated [41], alkenes are converted into the corresponding epoxides [42], carbon monoxide is oxidized to carbon dioxide [43], ammonia is oxidized to hydroxylamine [44], and some aromatic compounds and cyclic alkanes are hydroxylated [45]. To determine the “q” value, the user must establish if the reaction is mono-oxygenase-catalyzed or di-oxygenase-catalyzed. But in the most common cases, the “q” value ranges from 0 to 4. For a detailed description of this procedure the reader can refer to Xiao and VanBriesen (2006, 2008) [13], [14].

### 3.2 Case-studies

Some different microbial processes have been selected to demonstrate the potential and versatility of the MbT-Tool showing the output of the simulator, which is a set of MMRs with the corresponding yield prediction value.

In the experiments carried out by Battley (2013, 2007, 1995) [32], [35], [36] related to the *Saccharomyces cerevisiae*, he has established C_6.33_H_10.21_O_3.53_N as the empirical chemical formula for this yeast. Considering this information, using the MbT-Tool it is possible to represent two metabolic pathways: the biomass synthesis from glucose, and the biomass synthesis from pyruvate to ethanol. Before executing the MbT-Tool, it is necessary to determine some parameters. We used TEEM1 because we consider that there is no oxygenase reaction involved and the c-source is not a C1 compound. We used NH_4_^+^ because this chemical species is the universal N-source to biomass synthesis, and for *ε* we used a value that allows us to obtain the reported cell yield growing on glucose of the 0.098 mol Ccells/mol Cdonor [36]. Using the MbT-Tool with the established inputs, theoretical considerations and TEEM1 with *ε* value of 0.57 for the first pathway and *ε* value of 0.84 for the second one, the MMRs and the yields are:

Biomass synthesis from glucose:

C_6_H_12_O_6_ + 0.094 NH_4_^+^ + 2.25 HCO_3_^−^ → 0.094 C_6.33_H_10.21_O_3.53_N + 2.16 CH_3_COCOO^−^ + 1.17 CO_2_ + 3.59 H_2_O with an Yc/c = 0.099 mol Ccells/mol Cdonor.

Biomass synthesis from pyruvate to ethanol:

CH_3_COCOO^−^ + 0.047 NH_4_^+^ + 1.325 H_2_O → 0.047 C_6.33_H_10.21_O_3.53_N + 0.734 CH_3_CH_2_OH + 0.283 CO_2_ + 0.953 HCO_3_^−^ with an Yc/c = 0.098 mol Ccells/mol Cdonor.

In the published research carried out by H.W. van Verseveld [46], [47], [48] related to the growth of *Paracoccus denitrificans*, and considering succinate as electron donor and various final electron acceptors, the reported formula for this denitrifying bacterium was established as C_3_H_5.4_O_1.45_N_0.75_. To write MMRs, we considered succinate as C-source, NH_4_^+^ as N-source for biomass synthesis and the main electron acceptors involved in the de-nitrification pathway. Taking into account this information we used the MbT-Tool with TEEM2 and different *ε* values to represent a sequence of four reduction reactions (NO_3_^−^ → NO_2_^−^ → NO → N_2_O → N_2_) using MMRs for this denitrifying bacterium.

First reaction (NO_3_^−^ → NO_2_^−^): (C_4_H_4_O_4_)^2^^−^ + 0.30 NH_4_^+^ + 4.55 NO_3_^−^ → 0.40 C_3_H_5.4_O_1.45_N_0.75_ + 4.55 NO_2_^−^ + 1.10 CO_2_ + 1.70 HCO_3_^−^ + 0.67 H_2_O. (*ε* = 0.41).

Second reaction (NO_2_^−^ → NO): (C_4_H_4_O_4_)^2^^−^ + 0.58 NH_4_^+^ + 4.55 NO_2_^−^ + 4.55 H^+^ → 0.77 C_3_H_5.4_O_1.45_N_0.75_ + 4.55 NO + 0.26 CO_2_ + 1.42 HCO_3_^−^ + 2.64 H_2_O. (*ε* = 0.84).

Third reaction (NO → N_2_O): (C_4_H_4_O_4_)^2^^−^ + 0.58 NH_4_^+^ + 4.55 NO → 0.77 C_3_H_5.4_O_1.45_N_0.75_ + 2.28 N_2_O + 0.26 CO_2_ + 1.42 HCO_3_^−^ + 0.36 H_2_O (*ε* = 0.56).

Final reaction (N_2_O → N_2_): (C_4_H_4_O_4_)^2^^−^ + 0.58 NH_4_^+^ + 2.28 N_2_O → 0.77 C_3_H_5.4_O_1.45_N_0.75_ + 2.28 N_2_ + 0.26 CO_2_ + 1.42 HCO_3_^−^ + 0.36 H_2_O. (*ε* = 0.53).

Using these MMRs we created an IBM called INDISIM-Paracoccus [27], [31], [48], [49], which is designed to study the de-nitrification process carried out by the bacteria *P. denitrificans* in order to explore the consequence of different priorities in the individual use of electron-acceptors on the denitrification pathway [27].

Battley (1987) [33] established C_3.85_H_6.69_O_1.78_N as the empirical chemical formula for *Escherichia coli*. Considering this information, using the MbT-Tool it is possible to represent its diauxic growth on glucose and lactose. With the obtained MMR, it is possible to begin a modelling project related to the quantitative determination of metabolic fluxes during co-utilization of two C-sources [49] or a modelling project related to the organization of metabolic reaction networks [50]. We used TEEM2 because we consider that at least one mono-oxygenase-catalyzed reaction is involved (q = 1) [13], [14]. We used NH_4_^+^ as N-source to biomass synthesis. We used two eDs (glucose and lactate) and one eA (oxygen), and for *ε* we used a 0.37 value [11]. Using the MbT-Tool with the established inputs, the MMRs and the yields are:

C_6_H_12_O_6_ + CH_3_CHOHCOO^−^ + 5.05 O_2_ + 1.018 NH_4_^+^ + 0.018 HCO_3_^−^ → 1.018 C_3.85_H_6.69_O_1.78_N + 5.097 CO_2_ + 7.133 H_2_O.

Bacterial yield: Yg/m = 97.139 (gramscells/moldonor); Yc/m = 3.917 (molCcells/moldonor) and Yc/c = 0.435 (molCcell/molCdonor).

**Fig. 4:**
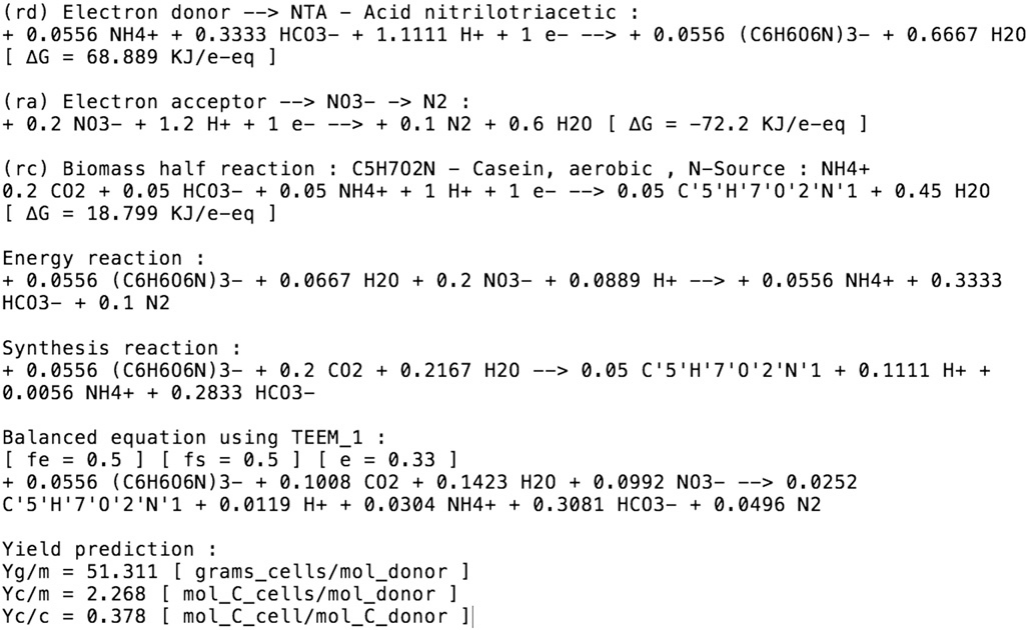
General outputs of MbT-Tool for the calculations related to the degradation of nitrilotriacetic acid in the absence of molecular oxygen (using nitrate as electron acceptor) by a gram-negative bacterium.

In the MbT-Tool, we selected a gram-negative bacterium with the empirical formula of C_5_H_7_O_2_N [5]. This bacterium degrades nitrilotriacetic acid (NTA) in the absence of molecular oxygen. To write the metabolic equation using nitrate as eA, NTA is used as the eD, and TEEM1 is used as the thermodynamic approach with an *ε* value of 0.33 [4], [8], [9], [11], [13], [14]. Wanner et al. [51], reported a cell yield growing on NTA equal to 50.760 g cells/mol NTA for this microbial process, while the bacterial yield prediction obtained with the MbT-Tool is 51.311 g cells/mol NTA. All outputs are presented in Fig. 4.

## 4 Final remarks

The main purpose of this contribution is to present the development of the MbT-Tool and make it available to a wide spectrum of readers, showing how this tool could be used in different research frameworks. This tool is a tangible way of achieving the compression of a thermodynamic application connected with microbial metabolism, therefore, it transfers a theoretical knowledge to a diverse range of applications of interest.

The MbT-Tool is, as far as we know, the only open-access and open-source software, that allows the writing of MMRs based on thermodynamic concepts. To use the MbT-Tool, non-expert knowledge about microbial metabolism is necessary, and only the most basic organic and inorganic chemistry is enough. The MbT-Tool by itself does not simulate any microbiological, ecological or biotechnological process, but the results obtained from the MbT-Tool allow the user to start with the calculations or the simulations for the study of any of these research fields.

In addition, the two strongest key points of this tool include: i) that the user doesn't have to pay for using the MbT-Tool, and ii) that the user is able to modify the source-code to extend the scope of the MbT-Tool into their specific research field and expertise.

Moreover, the scope of the MbT-Tool is not limited to the number of the pre-programmed half-reactions included in this version. With the combination of these half-reactions, it is possible to write MMRs for diverse microbial functional groups, such as aerobic heterotrophs, nitrifiers, denitrifiers, methanogens, sulphate reducers, sulphide oxidizers and fermenters (in supplementary material, Table S6). Also, the power and scope of the MbT-Tool increases if we take into account the possibility of changing the molar relationship between the main four elements to define the empirical chemical formula that represents the microbial biomass.

We consider that results obtained through the MbT-Tool could be the starting point to deal with a modelling project in the framework of microbial ecology or bio-technological processes. The non-expert user achieves an MMR in which one of the products is the chemical composition of the micro-organism involved in the process and some values of its bacterial yield. With these reactions, the user can i) start to construct a model, ii) incorporate the information into an existing model or iii) start the calculations of the mass balance for a bioreactor.

However, this tool has a limitation: it is not possible to compare the MbT-Tool's outputs directly with experimental results. We consider that only after the construction of a model with the MbT-Tool's results would the non-expert user be able to interpret its results. A non-expert user is considered here as a person whose expertise is not in the field of non-equilibrium thermodynamics, and therefore, to develop and apply all the conceptual elements involved in this thermodynamic approach for the construction of living models could be a time-consuming task. In contrast, a person with experience in these MMRs could have enough criteria to use and interpret the output reactions from MbT-Tool in his academic or research context.

In the research field on IBMs, using the INDISIM methodology as a core model, for instance [24], [25], [26], [52], [53], [54], we realized that is essential to know the metabolic reactions carried out by the micro-organisms to increase or decrease their own biomass. These metabolic reactions could be used to design the individual metabolism model as well as to parameterize the IBM models. In this sense, we think that the MbT-Tool's outputs are a convenient means to advance with this type of model [27], [31], [55].

Finally, we consider that the user could take advantage of the MMRs provided by the MbT-Tool in a wide range of academic or research fields, such as, for example: i) as a source to design processes that take advantage of the microbial system [4], and (ii) as the bedrock to make a connection between the microbial biomass and the substrates used by the micro-organism for pollution control [10], [56]. Additionally, the NetLogo's rather flat learning curve and comprehensive documentation [57] make this a user-friendly tool, easily accessible to chemists, biologists, ecologists, engineers and modellers, among others. Users without extensive programming experience can modify the code, introducing new chemical species to write new reduction-half-reactions, as well as other options for investigating alternative metabolic pathways or adapting certain processes according to specific studies.

In conclusion, the description of the cellular metabolism by means of thermodynamic concepts is strategic for investigating microbial activity and modelling bio-technological processes. We are convinced that the MbT-Tool will facilitate users to think about the biochemistry of metabolism due to its simplicity of use, and its results could be an interesting starting point for a microbial modelling approach.

## Appendix A Supplementary data

Supplementary material Image 2

MbT-Tool. Image 3
