# Supplementary material for: MbT-Tool: An open-access tool based on Thermodynamic Electron Equivalents Model to obtain microbial-metabolic reactions to be used in biotechnological process
Source: Comput Struct Biotechnol J. 2016 Aug 26;14:325–32. doi: 10.1016/j.csbj.2016.08.001 (PMC5013251; doi:10.1016/j.csbj.2016.08.001)
Supplement: Supplementary file 1 — Supplementary material [file mmc1.docx]

| **Supplementary Material**  **Table S1.** Organic half-reactions and their standard Gibbs free energy (P=1 atm, t=25ºC and pH=7) according to Rittmann and McCarty (2001). | | | |
| --- | --- | --- | --- |
| **#** | **Name** | **Half-reaction** | **∆G^o^**  **(kJ/e^-^eq)** |
| O-1 | Acetate | ^1^/_8_ CO_2_ + ^1^/_8_ HCO_3_^-^ + H^+^ + e^-^ → ^1^/_8_ CH_3_COO^-^ + ^3^/_8_ H_2_O | 27.40 |
| O-2 | Alanine | ^1^/_6_ CO_2_ + ^1^/_12_ HCO_3_^-^ + ^1^/_12_ NH_4_^+^ + ^11^/_12_ H^+^ + e^-^ → ^1^/_12_ CH_3_CHNH_2_COO^-^ + ^5^/_12_ H_2_O | 31.37 |
| O-3 | Benzoate | ^1^/_5_ CO_2_ + ^1^/_30_ HCO_3_^-^ + H^+^ + e^-^ → ^1^/_30_ C_6_H_5_COO^-^ + ^13^/_30_ H_2_O | 27.34 |
| O-4 | Citrate | ^1^/_6_ CO_2_ + ^1^/_6_ HCO_3_^-^ + H^+^ + e^-^ → ^1^/_18_ (COO^-^)CH_2_COH(COO^-^) CH_2_COO^-^ + ^4^/_9_ H_2_O | 33.08 |
| O-5 | Ethanol | ^1^/_6_ CO_2_ + H^+^ + e^-^ → ^1^/_12_ CH_3_CH_2_OH + ^1^/_4_ H_2_O | 31.18 |
| O-6 | Formate | ^1^/_2_ HCO_3_^-^ + H^+^ + e^-^ → ^1^/_2_ HCOO^-^ + ^1^/_2_ H_2_O | 39.19 |
| O-7 | Glucose | ^1^/_4_ CO_2_ + H^+^ + e^-^ → ^1^/_24_ C_6_H_12_O_6_ + ^1^/_4_ H_2_O | 41.35 |
| O-8 | Glutamate | ^1^/_6_ CO_2_ + ^1^/_9_ HCO_3_^-^ + ^1^/_18_ NH_4_^+^ + H^+^ + e^-^ → ^1^/_18_ COOHCH_2_CH_2_CHNH_2_COO^-^ + ^4^/_9_ H_2_O | 30.93 |
| O-9 | Glycerol | ^3^/_14_ CO_2_ + H^+^ + e^-^ → ^1^/_14_ CH_2_OHCHOHCH_2_OH + ^4^/_9_ H_2_O | 38.88 |
| O-10 | Glycine | ^1^/_6_ CO_2_ + ^1^/_6_ HCO_3_^-^ + ^1^/_6_ NH_4_^+^ + H^+^ + e^-^ → ^1^/_6_ CH_2_NH_2_COOH + ^1^/_2_ H_2_O | 39.80 |
| O-11 | Lactate | ^1^/_6_ CO_2_ + ^1^/_12_ HCO_3_^-^ + H^+^ + e^-^ → ^1^/_12_ CH_3_CHOHCOO^-^ + ^1^/_3_ H_2_O | 32.29 |
| O-12 | Methane | ^1^/_8_ CO_2_ + H^+^ + e^-^ → ^1^/_8_ CH_4_ + ^1^/_4_ H_2_O | 23.53 |
| O-13 | Methanol | ^1^/_6_ CO_2_ + H^+^ + e^-^ → ^1^/_6_ CH_3_OH + ^1^/_6_ H_2_O | 36.84 |
| O-14 | Palmitate | ^15^/_19_ CO_2_ + ^1^/_92_ HCO_3_^-^ + H^+^ + e^-^ → ^1^/_92_ CH_3_(CH_2_)_14_COO^-^ + ^31^/_92_ H_2_O | 27.26 |
| O-15 | Propionate | ^1^/_7_ CO_2_ + ^1^/_14_ HCO_3_^-^ + H^+^ + e^-^ → ^1^/_14_ CH_3_ CH_2_COO^-^ + ^5^/_14_ H_2_O | 27.63 |
| O-16 | Pyruvate | ^1^/_5_ CO_2_ + ^1^/_10_ HCO_3_^-^ + H^+^ + e^-^ → ^1^/_10_ CH_3_ COCOO^-^ + ^2^/_5_ H_2_O | 35.09 |
| O-17 | Succinate | ^1^/_7_ CO_2_ + ^1^/_7_ HCO_3_^-^ + H^+^ + e^-^ → ^1^/_14_ (CH_2_)_2_(COO^-^)_2_ + ^3^/_7_ H_2_O | 29.09 |

| **Table S2.** Inorganic half-reactions and their standard Gibbs free energy (P=1 atm, t=25ºC and pH=7) according to Rittmann and McCarty (2001). | | |
| --- | --- | --- |
| **#** | **Half-reaction** | **∆G^o^**  **(kJ/e^-^eq)** |
| I-1 | ^1^/_8_ NO_3_^-^ + ^5^/_4_ H^+^ + e^-^ → ^1^/_8_ NH_4_^+^ + ^3^/_8_ H_2_O | - 35.11 |
| I-2 | ^1^/_6_ NO_2_^-^ + ^4^/_3_ H^+^ + e^-^ → ^1^/_6_ NH_4_^+^ + ^1^/_3_ H_2_O | - 32.93 |
| I-3 | ^1^/_6_ N_2_ + ^4^/_3_ H^+^ + e^-^ → ^1^/_3_ NH_4_^+^ | 26.70 |
| I-4 | Fe^3+^ + e^-^ → Fe^2+^ | - 74.27 |
| I-5 | H^+^ + e^-^ → ½ H_2_ | 39.87 |
| I-6 | ½ NO_3_^-^ + H^+^ + e^-^ → ½ NO_2_^-^ + ½ H_2_O | - 41.65 |
| I-7 | ^1^/_3_ NO_3_^-^ + ^4^/_3_ H^+^ + e^-^ → ^1^/_3_ NO + ^2^/_3_ H_2_O | - 39.00 |
| I-8 | ^1^/_4_ NO_3_^-^ + ^5^/_4_ H^+^ + e^-^ → ^1^/_8_ N_2_O + ^5^/_8_ H_2_O | - 57.54 |
| I-9 | ^7^/_24_ NO_3_^-^ + ^31^/_24_ H^+^ + e^-^ → ^1^/_6_ NO + ^1^/_16_ N_2_O + ^31^/_48_ H_2_O | - 48.27 |
| I-10 | ^1^/_5_ NO_3_^-^ + ^6^/_5_ H^+^ + e^-^ → ^1^/_10_ N_2_ + ^3^/_5_ H_2_O | - 72.20 |
| I-11 | 2H^+^ + NO_2_^-^ + e^-^ → NO + H_2_O | - 33.72 |
| I-12 | ^1^/_5_ NO_2_^-^ + ^4^/_3_ H^+^ + e^-^ → ^1^/_6_ N_2_ + ^2^/_3_ H_2_O | - 92.56 |
| I-13 | H^+^ + NO + e^-^ → ½ N_2_O + ½ H_2_O | - 115.83 |
| I-14 | H^+^ + ½ N_2_O + e^-^ → ½ N_2_ + ½ H_2_O | - 133.47 |
| I-15 | ^1^/_8_ SO_4_^2-^ + ^19^/_16_ H^+^ + e^-^ → ^1^/_16_ H_2_S + ^1^/_16_ HS^-^ + ^1^/_2_ H_2_O | 20.85 |
| I-16 | ^1^/_6_ SO_3_^2-^ + ^5^/_4_ H^+^ + e^-^ → ^1^/_12_ H_2_S + ^1^/_12_ HS^-^ + ^1^/_2_ H_2_O | 11.03 |
| I-17 | ^1^/_2_ SO_4_^2-^ + H^+^ + e^-^ → ^1^/_2_ SO_3_^2-^ + ^1^/_2_ H_2_O | 50.30 |
| I-18 | ^1^/_6_ SO_4_^2-^ + ^4^/_3_ H^+^ + e^-^ → ^1^/_6_ S + ^2^/_3_ H_2_O | 19.15 |
| I-19 | ^1^/_4_ SO_4_^2-^ + ^5^/_4_ H^+^ + e^-^ → ^1^/_8_ S_2_O_3_^2-^ + ^5^/_8_ H_2_O | 23.58 |
| I-20 | ¼ O_2_ + H^+^ + e^-^ → ½ H_2_O | - 78.72 |

| **Table S3**. Empirical chemical formulas of some microbes. Adapted from Rittmann and McCarty (2001). | | | |
| --- | --- | --- | --- |
| **#** | **Empirical formula** | **Growth substrate and environmental conditions** | **Reference** |
|  | ***Pure cultures*** |  |  |
| M-1 | C_5_H_8_O_2_N | Bacteria, acetate, aerobic | (Bailey and Ollis, 1986) |
| M-2 | C_5_H_8.33_O_0.81_N | Bacteria, undefined |  |
| M-3 | C_4_H_8_O_2_N | Bacteria, undefined |  |
| M-4 | C_4.17_H_7.42_O_1.38_N | *Aerobacter aerogenes*, undefined |  |
| M-5 | C_4.54_H_7.91_O_1.95_N | *Klebsiella aerogenes,* glycerol*,*  µ=0.1 h^-1^ |  |
| M-6 | C_4.17_H_7.21_O_1.79_N | *Klebsiella aerogenes,* glycerol*,*  µ=0.85 h^-1^ |  |
| M-7 | C_4.16_H_8_O_1.25_N | *Escherichia coli,* undefined | (Battley, 1993) |
| M-8 | C_3.85_H_6.69_O_1.78_N | *Escherichia coli,* glucose | (Battley, 1987) |
| M-9 | C_6.33_H_10.21_O_3.53_N | *Saccharomyces cerevisiae,* glucose | (Battley, 2013) |
| M-10 | C_4_H_7.2_O_1.93_N | *Paracoccus denitrificans,* succinate | (van Verseveld et al., 1983) |
| M-11 | C_5_H_9_O_2.5_N | *Agrobacterium tumefaciens,* succinate | (Kampschreur et al., 2012) |
| M-12 | C_4.17_H_8_O_1.75_N | Bacteria, undefined | (Holt et al., 1994) |
|  | ***Mixed cultures*** |  |  |
| M-13 | C_5_H_7_O_2_N | Casein, aerobic | (Porges et al., 1956) |
| M-14 | C_7_H_12_O_4_N | Acetate, ammonia N-source, aerobic | (Symons and McKinney, 1958) |
| M-15 | C_9_H_15_O_5_N | Acetate, nitrate N-source, aerobic |  |
| M-16 | C_9_H_16_O_5_N | Acetate, nitrite N-source, aerobic |  |
| M-17 | C_4.9_H_9.4_O_2.9_N | Acetate, methanogenic | (Speece and McCarty, 1964) |
| M-18 | C_4.7_H_7.7_O_2.1_N | Octanoate, methanogenic |  |
| M-19 | C_4.9_H_9_O_3_N | Glycine, methanogenic |  |
| M-20 | C_5_H_8.8_O_3.2_N | Leucine, methanogenic |  |
| M-21 | C_4.1_H_6.8_O_2.2_N | Nutrient broth, methanogenic |  |
| M-22 | C_5.1_H_8.5_O_2.5_N | Glucose, methanogenic |  |
| M-23 | C_5.3_H_9.1_O_2.5_N | Starch, methanogenic |  |
| M-24 | C_n_H_a_O_b_N_c_ | Bacteria, generic | ------ |

| **Table S4**. Cell synthesis half-reaction (*Rc*) with different N-sources. Adapted from Rittmann and McCarty (2001). | |
| --- | --- |
| **N-Source** | **Half-reaction (*Rc*)** |
| NH_4_^+^ | $\frac{\left( n-c \right)}{d} CO_{2}+ \frac{c}{d} NH_{4}^{+}+\frac{c}{d} HCO_{3}^{-}+H^{+}+ e^{-}= \frac{1}{d}C_{n}H_{a}O_{b}N_{c}+ \frac{(2n-b+c)}{d} H_{2}O$ $where, d=(4n+a-2b-3c)$ |
| NO_3_^-^ | $\frac{n}{d} CO_{2}+ \frac{c}{d} NO_{3}^{-}+\frac{\left( 4n+a-2b+6c \right)}{d}H^{+}+ e^{-}= \frac{1}{d}C_{n}H_{a}O_{b}N_{c}+ \frac{\left( 3c+2n-b \right)}{d} H_{2}O$  $where, d=(4n+a-2b+5c)$ |
| NO_2_^-^ | $\frac{n}{d} CO_{2}+ \frac{c}{d} NO_{2}^{-}+\frac{\left( 4n+a-2b+4c \right)}{d}H^{+}+ e^{-}= \frac{1}{d}C_{n}H_{a}O_{b}N_{c}+ \frac{(2c+2n-b)}{d} H_{2}O$  $where, d=(4n+a-2b+3c)$ |
| N_2_ | $\frac{n}{d} CO_{2}+ \frac{c}{2d} N_{2}+H^{+}+ e^{-}= \frac{1}{d}C_{n}H_{a}O_{b}N_{c}+ \frac{(2n-b)}{d} H_{2}O$  $where, d=(4n+a-2b)$ |

| **Table S5.** TEEM1 (Rittmann and McCarty, 2001) and TEEM2 (McCarty, 2007) core equations. | |
| --- | --- |
| **TEEM1** | **TEEM2** |
| $A=-\frac{\Delta G_{s}}{\varepsilon\Delta G_{e}}=-\frac{\frac{\left( \Delta G_{in}-\Delta G_{d} \right)}{\varepsilon^{n}}+\frac{\Delta G_{pc}}{\varepsilon}}{\varepsilon\left( \Delta G_{a}-\Delta G_{d} \right)}= \frac{f_{e}^{o}}{f_{s}^{o}}$ | $A=-\frac{\Delta G_{s}}{\varepsilon\Delta G_{e}}=-\frac{\frac{\left( \Delta G_{fa}-\Delta G_{d} \right)}{\varepsilon^{m}}+\frac{\left( \Delta G_{in}-\Delta G_{fa} \right)}{\varepsilon^{n}}+\frac{\Delta G_{pc}}{\varepsilon}}{\varepsilon\left( \Delta G_{a}-\Delta G_{d}-\frac{q}{p}\Delta G_{xy} \right)}= \frac{f_{e}^{o}}{f_{s}^{o}}$ |
|  |  |
| *∆G_in_* = Reduction potential for Pyruvate half-reaction (35.09 kJ/eeq). | *∆G_in_* = Reduction potential for Acetyl-CoA half-reaction (30.9 kJ/eeq). |
|  | *∆G_fa_* = Reduction potential for formaldehyde half-reaction (46.53 kJ/eeq for C1 compounds, 0 for others). |
|  | *∆G_xy_* = Reduction potential for NADH oxidation (219.2 kJ/mol). |
|  | *m* = +1 if *∆G_fa_* > 0, otherwise = *n*. |
|  | *p* = Number of electron equivalents per mole of substrate from half-reaction reduction equation. |
|  | *q* = Number of oxygenase reactions per mole substrate. |
| fs^o^ = Fraction of electron-donor electrons converted for synthesis (eeq cells/eeq donor). | |
| fe^o^ = Fraction of electron-donor electrons used for energy and converted to reaction products (eeq products/eeq donor) | |
| *∆G_pc_* = Gibbs free energy for intermediate conversion to cells (kJ/eeq) = 3.33 kJ/gcells (Molecular weight Cells/pcells) = 3.33(113/20) = 18.8 kJ/eeq with ammonia as nitrogen source and cell formulation of C_5_H_7_O_2_N. With nitrate, nitrite, or N_2_ as nitrogen source, pcells equals 28, 26 and 23 kJ/eeq, respectively (Rittmann and McCarty, 2001). | |
| *ε* = Energy transfer efficiency. | |
| *∆G_e_* = Gibbs free energy for energy reaction (kJ/eeq). | |
| *∆G_s_* = Gibbs free energy for cell synthesis reaction (kJ/eeq). | |
| *∆G_d_* = Reduction potential for electron donor half-reaction (kJ/eeq). | |
| *∆G_a_* = Reduction potential for electron acceptor half-reaction (kJ/eeq). | |
| *n* = +1 if *m* = *n* and *(∆G_in_* - *∆G_d_*) > 0, otherwise *n* = -1. | |
| *γ_d_* = Degree of reduction of electron donor. | |
| *γ_x_* = Degree of reduction of cells. | |
| Yc/c = Maximum bacterial yield (molC_mic_/molC_substrate_). | |

| **Table S6**. Microbial types taking into account the electron donor, the electron acceptor. Adapted from Rittmann and McCarty (2001). | | |
| --- | --- | --- |
| **Microbial Group** | **Electron donor** | **Electron acceptor** |
| Aerobic Heterotrophs | Organic half-reaction | O_2_ |
| Nitrifiers | NH_4_^+^  NO_2_^-^ | O_2_  O_2_ |
| Denitrifiers | Organic half-reaction  H_2_  S | NO_3_^-^, NO_2_^-^  NO_3_^-^, NO_2_^-^  NO_3_^-^, NO_2_^-^ |
| Methanogens | H_2_ | CO_2_ |
| Sulfate reducers | Acetate  H_2_ | SO_4_^2-^  SO_4_^2-^ |
| Sulfide oxidizers | H_2_S | O_2_ |
| Fermenters | Organic half-reaction | Organic half-reaction |

| **Table S7.** Column positions of chemical species on the matrix *reactions* in the computer code. | | | |
| --- | --- | --- | --- |
| **Column** | **Chemical Specie** | **Column** | **Chemical Specie** |
| 1 | O_2_ | 21 | HS^-^ |
| 2 | H^+^ | 22 | S_2_O_3_^2-^ |
| 3 | e^-^ | 23 | S |
| 4 | CO_2_ | 24 | Alanine |
| 5 | NH_4_^+^ | 25 | Benzoate |
| 6 | HCO_3_^-^ | 26 | ∆G |
| 7 | Biomass | 27 | Citrate |
| 8 | H_2_O | 28 | Ethanol |
| 9 | Acetate | 29 | Formate |
| 10 | NO_3_^-^ | 30 | Glucose |
| 11 | NO_2_^-^ | 31 | Glutamate |
| 12 | NO | 32 | Glycerol |
| 13 | N_2_O | 33 | Glycine |
| 14 | N_2_ | 34 | Lactate |
| 15 | H_2_ | 35 | Methane |
| 16 | Fe^3+^ | 36 | Methanol |
| 17 | Fe^2+^ | 37 | Palmitate |
| 18 | SO_4_^2-^ | 38 | Propionate |
| 19 | SO_3_^2-^ | 39 | Pyruvate |
| 20 | H_2_S | 40 | Succinate |

## Program and computer code

MbT-tool is programed using built-in language primitives and the extension “matrix” of NetLogo. The code contains all the instructions and mathematical formulas to write microbial metabolic reactions. When the user clicks on the button *calculate*, the first procedure that the MbT-tool executes is the creation of a square matrix of i-rows by i-columns called “Reactions”. The number (i) of rows and columns is the same and represents the number of chemical species programmed in the source code of the tool. Each row stores all numerical coefficients of each reduction-half-reaction with its Gibb’s standard free energy. Therefore each chemical species uses a different column (Table S7) and each half-reaction uses a different row. In all cases (except Gibb’s standard free energy), if the numerical coefficient is positive it means *reaction reactant* and if it is negative it means *reaction product*.

When the matrix *reactions* is ready, MbT-tool selects from its rows the electron donor (*Rd)* and the electron acceptor (*Ra)* taking into account the user selection of these half-reactions, and places the corresponding information in different matrices (the matrix called “Rd” for *Rd* and the matrix called “Ra” for *Ra*). In the case of *Rd* it is also established what chemical compound is the electron source, and this last information will be used in the procedure called *yield prediction*. With the matrices Rd and Ra ready, MbT-tool sets up *Rc* taking into account the user selection of the microorganism empirical chemical formula and the N-source. This information is stored in a matrix called “b-biomass”. The next step is to make the thermodynamic calculations following the mathematical expressions of TEEM (taking into account the user selection between TEEM1 or TEEM2). If the TEEM2 model is selected, MbT-tool uses a value of the parameter “q” that represents the number of oxygenase reactions per mole of substrate (the default value for “q” is zero).

In the thermodynamic calculations, the Gibb’s free energy for *Rc* (∆G_pc_) is evaluated depending on the N-source and the estimated value of 3.33 kJ per gram cell (McCarty, 1971; Rittmann and McCarty, 2001). This value is referred to the empirical cell formula of C_5_H_7_O_2_N (Rittmann and McCarty, 2001) and MbT-tool adjusts it depending on the user selection of the micro-organism empirical chemical formula.

The label to mark each empirical chemical formula presented in Table S3 is made with two words, the first one indicates the type or name of the micro-organism and the second one indicates the substrate where the experiments for the microbial growth took place. But in any case this nomenclature conditions the normal operation of MbT-tool, because the information valid to make the calculations is only the molar relationship between the four main elements (C, H, O, N).

To complete the information MbT-tool uses the value of *ε* selected by the user. With all this information, MbT-tool displays the half-reactions of *Rd* and *Ra* with their Gibb’s free energy, the *Rc*, *Re*, *Rs*, the portion of electrons (*fe^o^*) to generate energy, the portion of electrons (*fs^o^*) for cell synthesis, the energy-transfer-efficiency, the balanced equation (*R*) in which the micro-organisms responsible for the process are included, and also the values of microbial yield prediction using the units g_cells_ mol_donor_^-1^, molC_cells_ mol_donor_^-1^ and molC_cells_ molC_donor_^-1^. MbT-tool allows the user to download the displayed results or outputs generated when the button called *download* is used.

***How to add others half-reactions for electron donor and electron acceptor?***

The user could add another half-reaction using the chemical species programmed just by adding another row in the matrix *reactions*. It is also possible to add another chemical specie just by adding another column to the matrix *reactions*. In the case that a new chemical specie is added, its necessary to add another reduction-half-reaction.

As an example of this procedure we will present the steps to add new chemical specie (NTA – Acid nitrilotriacetic) and it’s half-reaction into the source code of MbT-Tool. The user must follow the following steps to do it:

- Modify (increase) the number called “column_matrix”: this indicates the number of chemical species programmed in the current version of MbT-tool. With the changes to this number the user modifies the number of rows and columns of the matrix *reactions*.

According to Table S7 the number of chemical species programed is forty, due to we want to add a new one; this number will be increased in one unit. In the procedure *setup-reactions*, the NetLogo code will be:

set column_matrix 41

- Write the new half-reaction according to MbT-Tool’s format (if the stoichiometric coefficient is positive it means *reaction reactant* and if it is negative it means *reaction product*) and the positions of the matrix *reactions* showed in table S7.

The half-reaction for NTA is:

^1^/_18_ NH_4_^+^ + ^6^/_18_ HCO_3_^-^ + ^20^/_18_ H^+^ + 1 e- → ^1^/_18_ (C_6_H_6_O_6_N)^3-^ + ^12^/_18_ H2O ∆Gº = 68.889 KJ/e-eq

In the procedure *setup-reactions*, the NetLogo code will be:

matrix:set reactions 37 1 (20 / 18) ; H+

matrix:set reactions 37 2 (1) ; e-

matrix:set reactions 37 4 (1 / 18) ; NH4+

matrix:set reactions 37 5 (6 / 18) ; HCO3-

matrix:set reactions 37 7 (-12 / 18) ; H2O

matrix:set reactions 37 40 (-1 / 18) ; NTA - Acid nitrilotriacetic

matrix:set reactions 37 25 (68.889) ; ∆G

Each line of this code have especial structure: first, the NetLogo primitive “matrix:set”, followed by the name of the matrix, in our case *reactions*, followed by a number which indicates the row of the matrix where will be added the half-reaction, followed by a number which indicates the column position for the stoichiometric coefficient according to table S7, followed by a number between parenthesis, this number is the stoichiometric coefficient of the chemical specie in the reduction-half reaction (positive = reactant and negative = product, only in the case of the standard Gibbs energy this rule do not apply, column 25 in matrix *reactions*), and finally a comment with the name of the chemical specie.

- Add the new reaction name on the sliders to select “Electron_donor” and “Electron_acceptor”.

Due to the new chemical specie to be added is an organic one, the label for both sliders will be: “NTA – Acid nitrilotriacetic” into the label “organic reactions”.

- Add new lines of code in the section-code to define the electron donor and the electron acceptor.

In the case of the electron donor, into the procedure *setup-electron-donor*, the NetLogo code will be:

if Electron_donor = "NTA - Acid nitrilotriacetic" [set rd matrix:submatrix reactions 37 0 38 column_matrix set c 40]

The structure of this code shows us: first, the condition to choose the electron donor labeled, and second the commands to execute if the condition is accomplish.

Using the NetLogo’s primitive “matrix:submatrix” we define the matrix “rd” to storage the half-reaction to the electron donor, the numbers after the name of the source matrix (*reactions)* points out the row that will be taken from the matrix *reactions* according to the selection in the slider; finally the “c” value points out the position of this organic compound in the matrix *reaction* (procedure *setup-reaction*), this value is used into the procedure called *yield-prediction*.

In the case of the electron acceptor, into the procedure *setup-electron-acceptor*, the NetLogo code will be:

if Electron_acceptor = "NTA - Acid nitrilotriacetic" [set ra matrix:submatrix reactions 37 0 38 column_matrix]

The structure of this code is exactly the same than the electron donor, but in the case of the electron acceptor we define the matrix “ra” to storage the half-reaction for it.

- Add new lines of code in the procedure called “output_all”.

The procedure *output_all* allows MbT-Tool to display its results. Therefore, the user has to add two new lines of code, one line to reactants and one line to products. The code for reactant will be:

ifelse (matrix:get to-print 0 40) <= 0 [ ][output-type "+ " output-type precision (matrix:get to-print 0 40) 4 output-type " (C6H6O6N)3- "]

And the code for products will be:

ifelse (matrix:get to-print 0 40) >= 0 [ ][output-type "+ " output-type precision abs (matrix:get to-print 0 40) 4 output-type " (C6H6O6N)3- "]

The structure of this code follow the syntaxes for the NetLogo’s primitives: matrix:get, output-type and precision. The main difference is in the condition (<= 0 or >= 0) with this structure MbT-Tool established if the stoichiometric coefficient is a reactant or product.
